# Suspected native aortic valve thrombosis complicated by recurrent left main coronary artery embolism and acquired Gerbode defect: a case report

**DOI:** 10.1093/ehjcr/ytag447

**Published:** 2026-06-10

**Authors:** Gabriela Lipošćak, Nikola Kos, Krešimir Kordić, Lucija Svetina, Ozren Vinter

**Affiliations:** Department of Internal Medicine, General Hospital ‘Dr. Anđelko Višić ’, Antuna Mihanovića 8, Bjelovar 43000, Croatia; Department of Cardiology, Clinical Hospital Center Sisters of Charity, Vinogradska cesta 29, Zagreb 10 000, Croatia; Department of Cardiology, Clinical Hospital Center Sisters of Charity, Vinogradska cesta 29, Zagreb 10 000, Croatia; School of Medicine, Catholic University of Croatia, Ilica 244, Zagreb 10 000, Croatia; Department of Cardiac Surgery, University Hospital Center Zagreb, Kišpatićeva 12, Zagreb 10 000, Croatia; Department of Cardiology, Clinical Hospital Center Sisters of Charity, Vinogradska cesta 29, Zagreb 10 000, Croatia; School of Medicine, Catholic University of Croatia, Ilica 244, Zagreb 10 000, Croatia

**Keywords:** Native aortic valve thrombosis, Left main coronary artery embolism, Gerbode defect, Case report

## Abstract

**Background:**

Native aortic valve thrombosis (NAVT) is an uncommon cause of coronary embolism and myocardial infarction. In patients with severe aortic valve disease treated surgically, a post-operative communication between the left ventricle and right atrium (i.e. acquired Gerbode defect) is a rare complication.

**Case summary:**

We report the case of a 67-year-old man with suspected NAVT presenting with myocardial infarction and a post-operative acquired Gerbode defect. Coronary angiography showed a floating thrombus in the left main coronary artery without significant culprit-vessel atherosclerotic disease, suggesting coronary embolism. Multiple aspiration thrombectomies and glycoprotein IIb/IIIa inhibitor therapy were performed. Recurrent chest pain prompted repeat coronary angiography, which confirmed thrombus recurrence and resolution after repeat treatment. Intravascular ultrasound excluded significant culprit-vessel atherosclerotic disease. Aspirated material revealed a subacute thrombus with microcalcifications on histopathological examination. Echocardiography showed severe calcific aortic stenosis, a likely predisposing factor for NAVT. Atrial fibrillation had not been documented during the embolic event or previously and was only detected later during hospitalization, prompting initiation of anticoagulation (CHA2DS2-VASc score 3). Consequently, dual antiplatelet therapy was replaced with apixaban and ticagrelor until surgery. After clinical stabilization, the patient underwent surgical aortic valve replacement. No left atrial appendage thrombus was found intra-operatively after 3 months of anticoagulation. Post-operatively, multimodal imaging identified and quantified an acquired Gerbode defect. Given its haemodynamic insignificance, a conservative approach was adopted.

**Discussion:**

This case highlights underreported complications of severe aortic stenosis and its treatment, emphasizing the importance of multimodal imaging in the diagnosis of NAVT and post-operative Gerbode defect.

Learning points

- Native aortic valve thrombosis is a rare but important cause of acute myocardial infarction due to coronary embolism and should be considered in patients with severe native aortic valve disease.
- Acquired iatrogenic Gerbode defect is a rare complication after surgical aortic valve replacement and is frequently misdiagnosed as tricuspid regurgitation.

## Introduction

Native aortic valve thrombosis (NAVT) is a rare condition associated with aortic valve disease, aortic root pathology, or hypercoagulable states. The non-coronary cusp is most frequently involved, followed by the left coronary cusp. Clinical presentation ranges from asymptomatic disease (23%) to embolic events (59%) and heart failure (7%). Myocardial, limb, and cerebrovascular ischaemia are the most common embolic manifestations. In-hospital mortality of NAVT is estimated at 20%.^1^

Systematic diagnostic evaluation is pivotal for diagnosis confirmation. Transoesophageal echocardiography (TOE) and aortic root angiography show high diagnostic sensitivity, whereas transthoracic echocardiography (TTE) and invasive coronary angiography (ICA) sensitivities are estimated at 59% and 29%, respectively.^1^

In patients treated with surgical aortic valve replacement (SAVR) for severe aortic valve disease, a post-operative left ventricular-right atrial shunt [acquired Gerbode defect (AGD)] is an uncommon complication.^2^ Small shunts are usually asymptomatic, whereas large shunts may lead to right heart chamber enlargement and predominantly right-sided heart failure. Transoesophageal echocardiography is a high-sensitivity method for detecting LV-RA shunts, especially in patients with prosthetic heart valves. Acquired Gerbode defect must be distinguished from ruptured sinus of Valsalva aneurysm, ventricular septal defect, tricuspid regurgitation, and pulmonary arterial hypertension. Treatment decisions for AGD depend on shunt magnitude and symptoms. Asymptomatic or small defects can be managed conservatively.^3^

## Summary figure

**Table:** 

Time	Event
Day 0 (presentation)	Subacute NSTEMI; coronary angiography revealed a large mobile thrombus in the LMCA; manual aspiration thrombectomy performed; eptifibatide administered.
Day 1	Recurrent chest pain; repeat angiography confirmed recurrent LMCA thrombus extending into the ostium of the LCx; repeat aspiration and eptifibatide.
During index hospitalization	TTE confirmed severe calcific aortic stenosis with preserved LVEF and regional wall motion abnormalities; paroxysmal atrial fibrillation documented and treated; oral anticoagulation initiated.
Pre-operative work-up (1 month after discharge)	ECG-gated coronary CT performed; no delayed-phase imaging to definitively exclude LAA thrombus.
Operation (3 months after index hospitalization)	SAVR with a bioprosthesis, pulmonary vein isolation, and LAA occlusion; bicuspid aortic valve confirmed intra-operatively; no LAA thrombus identified after anticoagulation.
Post-operative	Echocardiography detected a LV-RA shunt consistent with acquired Gerbode defect; cardiac CT quantified a 3-mm defect; radionuclide ventriculography excluded haemodynamic significance; conservative management and follow-up.

## Case presentation

A 67-year-old man presented to the emergency department with chest pain due to subacute non-ST-segment elevation myocardial infarction. The patient’s medical history included congenital solitary kidney, arterial hypertension, dyslipidaemia, prior myocardial infarction (2021), and active smoking. Admission findings are summarized in *Table 1*.

**Table 1:** Admission findings

Parameter	Finding
Oxygen saturation	97% on room air
Blood pressure	120/70 mmHg
Killip class	Killip I; no signs of heart failure, normal haemodynamics
ECG	Sinus rhythm, 100 b.p.m.; ST-segment elevation in aVR; 1 mm ST-segment depression in V4-V6, I, II, and aVL
Cardiac biomarkers	High-sensitivity troponin I 7836 ng/L; Creatine kinase 815 U/L
Renal function	Creatinine 70 µmol/L; eGFR 93 mL/min/1.73 m^2^

Invasive coronary angiography revealed a large floating thrombus within the left main coronary artery (LMCA), with no significant atherosclerotic culprit-vessel disease and a moderate non-culprit lesion in the first diagonal branch. Repeat aspiration thrombectomy and eptifibatide (glycoprotein IIb/IIIa inhibitor) therapy were performed, with TIMI 3 flow preserved before and after the intervention.

**Figure 1:**
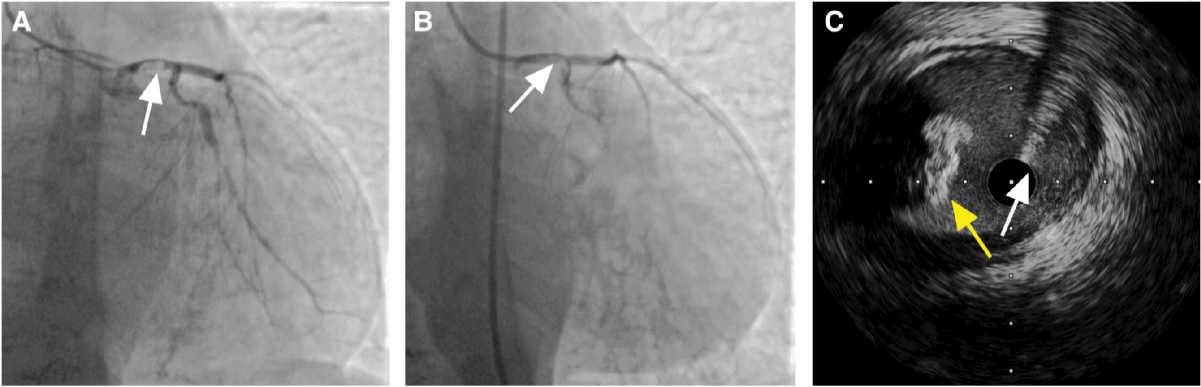
(*A*) Floating thrombus in left main coronary artery on initial invasive coronary angiography (arrow). (*B*) Recurrent thrombus in left main coronary artery/left circumflex artery ostium on repeat invasive coronary angiography (arrow). (*C*) Intravascular ultrasound showing non-obstructive, stable atherosclerotic plaque without evidence of rupture or erosion (yellow arrow) and partial thrombus resolution (white arrow).

The following day, recurrent chest pain prompted repeat ICA, which showed a partially obstructive LMCA thrombus extending into the left circumflex artery (LCx) ostium and was treated with repeat aspiration thrombectomy and eptifibatide. Invasive coronary angiography and intravascular ultrasound (IVUS) confirmed an adequate final result, with TIMI 3 flow in all vessels and no significant atherosclerotic disease or plaque disruption in the culprit vessels (*Figure 1*); therefore, LMCA stenting was deferred. Aspirated material showed a subacute thrombus with microcalcifications on histopathological examination.

**Figure 2:**
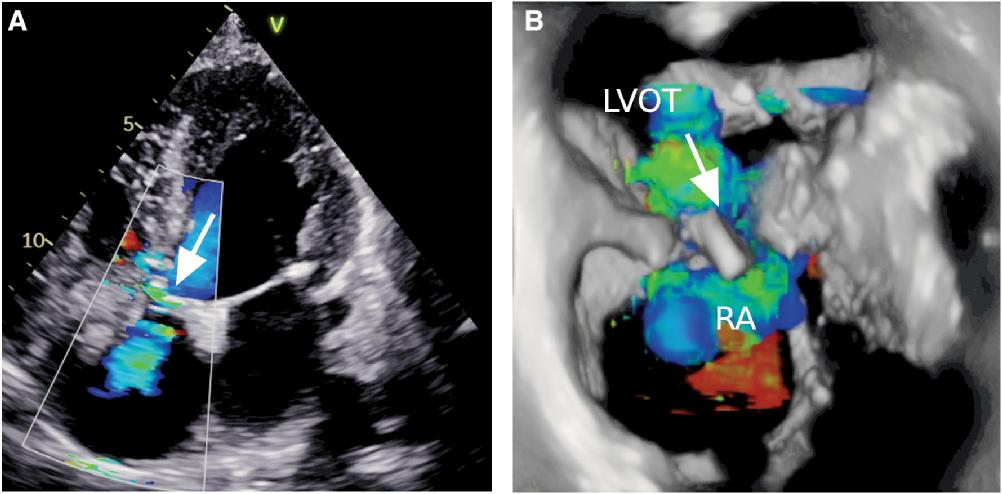
Acquired Gerbode defect (arrow) visualized on transthoracic echocardiography (*A*), transoesophageal echocardiography, and 3D reconstruction (*B*).

Transthoracic echocardiography revealed severe calcific aortic stenosis (Vmax 4.8 m/s, mean pressure gradient 56 mmHg), preserved left ventricular systolic function (LVEF 50%), and posterior and lateral wall hypokinesia. In-hospital paroxysmal atrial fibrillation (AF) onset warranted lifelong anticoagulation (CHA_2_DS_2_-VASc 3), leading to a change from aspirin-ticagrelor dual antiplatelet therapy for acute coronary syndrome to apixaban plus ticagrelor until surgery. Ticagrelor was maintained to reduce recurrent coronary thrombosis risk, while aspirin omission reduced bleeding risk per ACS guidelines.^4^ One month after discharge, pre-operative ECG-gated coronary CT angiography was concordant with ICA findings. No delayed-phase imaging was performed to definitively exclude left atrial appendage (LAA) thrombus. The patient underwent SAVR with a bioprosthesis, pulmonary vein isolation, and LAA occlusion 3 months after the index hospitalization. Surgery was planned earlier but was deferred due to pneumonia. Intra-operatively, the aortic valve was found to be bicuspid and no LAA thrombus was present after prolonged anticoagulation. In the post-operative course, paroxysmal AF was noted, and AGD was detected on echocardiography (*Figure 2*). Cardiac CT demonstrated a 3-mm defect. Radionuclide ventriculography showed a Qp/Qs of 1.26 indicating a small, haemodynamically insignificant left-to-right shunt. Absence of right ventricular dilatation, pulmonary hypertension, or clinical signs of right-sided volume overload supported this interpretation and favoured conservative management with follow-up.

## Discussion

Native aortic valve thrombosis is a rare clinical entity and a potential cause of coronary embolism.^1^ This case underscores the diagnostic and therapeutic challenges of LMCA thrombosis without significant culprit-vessel atherosclerosis, followed by a rare post-operative complication (AGD). In our case, the diagnostic reasoning was stepwise. First, the mechanism of LMCA obstruction was assessed as embolic rather than atherothrombotic. This conclusion was based on angiographic appearance of a large, mobile thrombus within the LMCA with TIMI 3 flow preservation, recurrent thrombus formation despite aspiration thrombectomy and eptifibatide, and IVUS showing no plaque rupture or plaque disruption in the culprit vessels. Second, potential embolic sources were considered, including the native aortic valve, LAA, and intracardiac shunts.^5^ Alternative causes of embolism, such as left ventricular aneurysm or mitral valve apparatus abnormality, were excluded by echocardiography. Paroxysmal AF is a plausible embolic source; however, the timing of AF detection, thrombus microcalcifications, and absence of LAA thrombus at surgery after prolonged anticoagulation make LAA embolism less likely, although not excluded.^6^ Third, microcalcifications on histopathological analysis of aspirated thrombus together with severe calcific aortic stenosis and the intra-operative finding of a bicuspid valve support a possible valvular origin of embolism, although this remains unproven.^7^ Transoesophageal echocardiography allows direct visualization of native aortic valve thrombus and exclusion of alternative embolic sources, including LAA thrombus or intracardiac shunts.^1,8-10^ In our case, omission of TOE was a deliberate management decision due to urgent coronary intervention and planned surgery. In addition, paroxysmal AF constituted an indication for anticoagulation (CHA_2_DS_2_-VASc score 3), making TOE unlikely to alter management. The absence of TOE and delayed-phase CT precludes definitive source identification; therefore, the diagnosis remains inferential. However, the available evidence may support a potential valvular source of embolism. High-sensitivity imaging for NAVT (i.e. TOE) may be useful in selected cases without an indication for anticoagulant or surgical treatment.

Therapeutically, this case highlights the value of IVUS on LMCA interventions, prompting a non-stenting strategy and multidisciplinary individualized management. Post-operatively, AGD required comprehensive diagnostic evaluation. Such defects are rare after SAVR and often misdiagnosed as tricuspid regurgitation. In this case, multimodal imaging, comprising transthoracic and TOE, cardiac CT, and radionuclide ventriculography, enabled reliable anatomical and functional assessment. Given the small defect and haemodynamic insignificance, a conservative strategy was adopted. Doppler echocardiography showed no change in shunt-related haemodynamics over 2 months.

In conclusion, NAVT should be considered in patients with severe aortic valve disease presenting with coronary embolism, even with AF. Integrating multimodal imaging and, when available, histopathological analysis may help establish the embolic mechanism. Awareness of post-operative complications such as AGD is important to guide management and avoid unnecessary interventions.

## Data Availability

The data underlying this article are not publicly available due to patient confidentiality.

## Lead author biography

Gabriela Lipošćak, MD, is a board-certified cardiologist working at General Hospital Bjelovar.

## Notes

**Consent:** Written informed consent was obtained from the patient for publication of this case and all accompanying images.

## References

[1] Alajaji W, Hornick JM, Malek E, Klein AL. The characteristics and outcomes of native aortic valve thrombosis: a systematic review. J Am Coll Cardiol 2021;78:811–824. 34412815 10.1016/j.jacc.2021.06.023

[2] Sunderland N, El-Medany A, Temporal J, Pannell L, Doolub G, Nelson M, et al The Gerbode defect: a case series. Eur Heart J Case Rep 2021;5:ytaa548. 33598621 10.1093/ehjcr/ytaa548 PMC7873810

[3] Saker E, Bahri GN, Montalbano MJ, Johal J, Graham RA, Tardieu GG, et al Gerbode defect: a comprehensive review of its history, anatomy, embryology, pathophysiology, diagnosis, and treatment. J Saudi Heart Assoc 2017;29:283–292. 28983172 10.1016/j.jsha.2017.01.006 PMC5623025

[4] Byrne RA, Rossello X, Coughlan JJ, Barbato E, Berry C, Chieffo A, et al 2023 ESC guidelines for the management of acute coronary syndromes. Eur Heart J 2023;44:3720–3826. 37622654 10.1093/eurheartj/ehad191

[5] Cozza E, Cito V, Giumbini G, Marsili G, Barletta M, Zannoni J, et al Cardioembolic sources and stroke prevention: a systematic review. Cardiovasc Ultrasound 2026;24:8. 41834030 10.1186/s12947-026-00367-5 PMC12990578

[6] Chatterjee NA, Lubitz SA. Systemic embolic events (SEE) in atrial fibrillation: SEEing embolic risk more clearly. Circulation 2015;132:787–789. 26224812 10.1161/CIRCULATIONAHA.115.018172 PMC4558335

[7] Chernysh IN, Nagaswami C, Kosolapova S, Peshkova AD, Cuker A, Cines DB, et al The distinctive structure and composition of arterial and venous thrombi and pulmonary emboli. Sci Rep 2020;10:5112. 32198356 10.1038/s41598-020-59526-x PMC7083848

[8] Yu S, Zhang H, Li H. Cardiac computed tomography versus transesophageal echocardiography for the detection of left atrial appendage thrombus: a systematic review and meta-analysis. J Am Heart Assoc 2021;10:e022505. 34796743 10.1161/JAHA.121.022505 PMC9075398

[9] Romero J, Husain SA, Kelesidis I, Sanz J, Medina HM, Garcia MJ. Detection of left atrial appendage thrombus by cardiac computed tomography in patients with atrial fibrillation: a meta-analysis. Circ Cardiovasc Imaging 2013;6:185–194. 23406625 10.1161/CIRCIMAGING.112.000153

[10] Pushparajah K . Non-invasive imaging in the evaluation of cardiac shunts for interventional closure. Front Cardiovasc Med 2021;8:651726. 34222361 10.3389/fcvm.2021.651726 PMC8253251

